# Learning about depression by watching gaming videos: a case study on the potential of digital games for psychoeducation and destigmatization

**DOI:** 10.3389/fpsyg.2025.1585571

**Published:** 2025-05-26

**Authors:** Marco Rüth, Raoul Bachmayer, Kai Kaspar

**Affiliations:** Department of Psychology, University of Cologne, Cologne, Germany

**Keywords:** digital games, depression, psychoeducation, stigmatization, learning motivation, narrative engagement, mixed methods

## Abstract

**Introduction:**

People with depression can also suffer from stigmatization related to depression. Psychoeducation is one way to alleviate stigmatization (destigmatization), so that particularly people without depression become more aware of depressive symptoms and can empathize better with depressed people. Here, we scrutinize the potential of digital games to support psychoeducation and destigmatization of depression.

**Methods:**

We conducted a mixed methods online study with 117 participants. In the intervention phase, participants watched gaming videos and noted down aspects that had left a lasting impression on them. In the evaluation phase, we used open-ended items and qualitative content analysis to investigate participants’ learning outcomes. We used quantitative scales to examine different facets of participants’ learning motivation, their narrative engagement, and destigmatization types.

**Results:**

Watching gaming videos resulted in several learning outcomes, including cognitive as well as emotional effects. Given a high general learning motivation regarding the topic depression and moderate narrative engagement in our sample, participants also reported strong conviction that such games can be an interesting and relevant medium to learn about depression. A multiple regression analysis revealed that 51% of variance in participants’ personal thoughts about depression could be explained by our proposed model. Males had a higher personal stigma than females. Personal stigma was also negatively related to participants’ depression literacy and their general learning motivation regarding the topic depression.

**Discussion:**

Our findings indicate that watching videos of digital games can support psychoeducation and destigmatization by reaching a broad audience and sensitizing people about mental illnesses such as depression.

## 1 Introduction

Depression is a mood disorder that can happen to everyone. Data from the Global Burden of Disease study indicate that 4% of the global population suffered from depression in 2021 (OWID, 2024). Further, major depression has been related to high suicide rates, illustrating the importance to raise awareness and educate people about depression (Fu et al., 2023). Indeed, common treatments of depression like pharmacotherapy or psychotherapy can be complemented by psychoeducation, which can reduce depressive symptoms and prevent depression in non-depressed and varied populations (Katsuki et al., 2022; Rigabert et al., 2020). Moreover, psychoeducation can help to reduce stigmatization (or promote destigmatization) of people with depressive symptoms and alleviate the related additional stress and discomfort. Stigmatization can be viewed from two sides: first, people can be stigmatized in terms of having an internalized, anticipated, and experienced stigma; second, people can stigmatize other people suffering from depressive symptoms by means of stereotypes, prejudices, and discrimination (Fox et al., 2018). The stigma of people with depressive symptoms is also known as self-stigma or internalized stigma, i.e., “the way in which people with mental health conditions see themselves as being mentally unwell and, therefore, of lesser value” (Thornicroft et al., 2022, p. 1442). Conversely, the public stigma is how “people in a given community or society views and acts toward people with mental health conditions” (Thornicroft et al., 2022, p. 1442).

One way to support psychoeducation and destigmatization are digital games that are perhaps rather known as popular entertainment tools. However, digital games have also been used to innovate education (e.g., Rüth and Kaspar, 2024; Wouters et al., 2013), to foster physical and mental health (e.g., Abd-Alrazaq et al., 2022; Rüth et al., 2023), and to reduce stigmatization related to mental illnesses (Rodríguez-Rivas et al., 2022). Still, digital games have yet been rarely used in destigmatization programs (Kerkemeyer et al., 2022), so that their potential and role in destigmatization require further investigation. Our aim is therefore to better understand the role of digital games in psychoeducation and destigmatization. More specifically, we focus on the potential of watching digital games (instead of actively playing), which also addresses people who do not play digital games themselves for various reasons (e.g., effort of playing digital games, lack of skills or access to digital games, or toxic online communities) but who would like to experience the special narrative engagement provided by digital games (Orme, 2022). Narration can be considered a key element of human communication as reflected in the term *homo narrans* (Fisher, 1985). How content is embedded in narratives or transported via them plays a key role in the effects of diverse media types on recipients’ engagement in and attitude toward the addressed topics (cf. Romney and Johnson, 2020; Zimmermann et al., 2025). Regarding psychoeducation, narrative texts were found to more effectively foster understanding and communication about mental health topics such as depression compared to cognitive learning questions regarding depression (Scholl et al., 2022). Further, the term *homo ludens* illustrates that gaming can also be considered a key human activity, whereas findings on learning with narrative games are rather ambivalent (cf. Rüth, 2025). Based on these theoretical and empirical considerations and since watching games has gained enormous importance and is of interest for a broad audience (Elliott, 2024; Hilvert-Bruce et al., 2018), this study focuses on examining the potential of watching gaming videos of a digital game about depression for psychoeducation and destigmatization.

### 1.1 Digital games and depression

Mental illnesses appear in many digital games, and depression is also represented in several popular digital games, including *Celeste, Detroit: Become Human, Gris, Kingdom Hearts III, Life is Strange*, and *The Awesome Adventures of Captain Spirit* (Kasdorf, 2023). However, it was found that depression and other mental illnesses are almost always represented in digital games in negative or stereotypical ways (Buday et al., 2022). While such negative representations may foster stigmatization, two experimental studies found that playing the digital game *Stigma-stop* significantly decreased the overall stigma related to mental health problems with small to medium effect sizes (Cangas et al., 2017, 2019). Another study on cognitive and social affordances of the digital games *Depression Quest* and *Actual Sunlight* found, based on community posts on the publishing platform *Steam*, that these games made players think about their own life and choices and could increase players’ understanding and empathy (Hoffman, 2019). Moreover, social affordances were expressed in the posts in terms of advice and encouragement for people with depression, experiences about how digital games were used to talk about depression, and that playing such digital games elicited the feeling that others also suffer from depression (Hoffman, 2019). It should be noted that some digital games may have potentials for relaxation but are not specifically designed to alleviate depressive symptoms and, therefore, playing such games may have no significant effects as indicated by an experimental study on the digital games *Journey* and *Flower* (Poppelaars et al., 2021). Further, narrative digital games in specific may have different effects on people, also depending on their individual characteristics such as learning motivation or prior knowledge as well as general phenomena of information processing (Rüth, 2025). So, some digital games can provide valuable tools for people with and without depressive symptoms, but the affordances and effects of games on various audiences need to be evaluated. Moreover, some digital games include several characters with mental illnesses or are not intended to educate about depression. Here, we focus on a digital game in which the main character suffers from depression and that was intentionally designed to sensitize the public for depression. Specifically, we evaluate the role of the narrative digital game *Duru* (Twisted Ramble Games, 2023) in psychoeducation and destigmatization of depression based on several research questions that we elaborate on in the following.

### 1.2 Different levels of learning about depression through digital games

The role of digital games in psychoeducation and destigmatization regarding mental health disorders such as depression can be understood in terms of different aspects.

First, digital games can enable perspective-taking and empathy with stigmatized people and lead to meaningful experiences that one can learn from and reflect on (cf. Daneels et al., 2023; Rüth and Kaspar, 2021). For instance, in the context of destigmatization, one can learn about the perspective of the stigmatized – in this case people with depressive symptoms – and reflect on stigmatization in terms of stereotypes, prejudices, and discrimination (Fox et al., 2018). In this study, we scrutinize what participants have learned from the digital game (RQ1a), what effects the digital game has had on the participants (RQ1b), and what effects participants think the digital game could have on others (RQ1c).

Second, to better understand the potential and practical significance of using digital games for psychoeducation and destigmatization, it is important to evaluate how motivated people are to learn with digital games about depression. Based on the ARCS-V model (Keller, 2016), which has been applied in digital games research (cf. Krath et al., 2021; Rüth and Kaspar, 2021), we focus on learning motivation in terms of participants’ conviction that digital games can be an interesting and relevant medium to learn about depression (RQ2a), their general learning motivation regarding the topic depression (RQ2b), and their satisfaction with the digital game at hand *(Duru)* as a learning medium (RQ2c).

Third, since *Duru* is a narrative digital game that contains a story about depression, we also evaluate participants’ engagement with the game’s narrative. In line with transportation theory (Green and Brock, 2000), narrative engagement is thought to be fundamental to understand the events, situations, and characters as well as to empathize with the characters’ emotions (Busselle and Bilandzic, 2009). Consequently, we here scrutinize to what extent participants paid attention to the game’s narrative, understood the game’s narrative, were emotionally engaged with the game’s narrative, and had the feeling of being in the narrated world (RQ3).

Fourth, we examine participants’ depression stigma in terms of what participants think about depression (*personal depression stigma*) and what participants think most other people would think about depression (*perceived depression stigma*). In the context of this study, we inspect how participants’ depression stigma is related to their learning motivation and narrative engagement regarding the digital game. Particularly participants’ personal depression stigma could be related to what they know about depression (*depression literacy*), as indicated by an online survey that found a negative correlation between high school and university students’ personal depression stigma and depression literacy (Al-Shannaq et al., 2023). Previous research also revealed mixed results on the role of age and gender in depression stigma (Al-Shannaq et al., 2023; Dietrich et al., 2014) and depression literacy (Al-Shannaq et al., 2023; Freitag et al., 2017). Taken together, we investigate how participants’ depression stigma is related to their learning motivation, narrative engagement, depression literacy, age, and gender (RQ4).

## 2 Methods

### 2.1 Participants

Sample size was determined based on the focal multiple linear regression model for personal depression stigma (RQ4). The minimum sample size required was *n* = 103, given seven independent variables in the model (see below) and a targeted medium-sized effect of *f^2^* = 0.15, a test power of 0.80, and a significance level of 0.05. A medium-sized effect was determined due to practical relevance and expected since previous work found that considering sociodemographic characteristics in a regression model for personal depression stigma can already result in a medium-sized effect (Griffiths et al., 2008). Overall, 134 participants provided informed consent and completed the study. Still, 17 participants were excluded who did not carefully complete the study (*n* = 3), did not fully watch the videos (*n* = 6), or did not pass the attention check described below (*n* = 8). Most participants have watched the videos in full screen (92.3%), and less than one in three participants have used headphones while watching (29.1%). However, these were no exclusion criteria since this may reflect individual usage preferences. The videos were displayed across the screen, and sound reproduction via loudspeakers suffices to follow the game’s narrative. Finally, we analyzed the data of 117 participants (81 female, 31 male, 5 diverse) aged 18–64 years (*M* = 26.49; *SD* = 10.44). Participants were recruited via the social media platforms Instagram, LinkedIn, Reddit, WhatsApp, and via mailing lists. An overview of sample characteristics can be found online in the Supplementary Table S1. Psychology students received 0.75 subject hours for their participation.

### 2.2 Design and procedure

We used a sequential mixed methods design and conducted an online study to provide results from an externally valid setting in which gaming videos are usually being watched. First, participants gave written informed consent and were informed that they will see sensitive content concerning depression. Participants were asked not to take part if they are undergoing psychotherapeutic treatment or are knowingly suffering from a mental illness, and they were informed about professional points of contact regarding mental health. Then, we instructed participants to pay attention to the following three videos, to watch them in a quiet environment and in full screen, to use headphones if possible, and to take notes on what effect the videos have on them. After each video, participants were asked to write down up to three aspects of the video that have left a lasting impression on them. Following the last video, participants were asked to name up to three aspects that they have learned by watching the videos (RQ1a), and what effects the digital game had on them (RQ1b) and could have on others (RQ1c). These responses also served as a filler task between the videos and the following attention check, which consisted of one dichotomous item per video about its content. Then, participants rated their learning motivation (RQ2), narrative engagement (RQ3), depression stigma (RQ4), and depression literacy (RQ4). Finally, participants provided sociodemographic information, indicated if they have completed the study carefully, and were debriefed in terms of information on the collected data and on professional points of contact regarding depression.

### 2.3 Materials

Participants watched three gaming videos of the 2D puzzle adventure game *Duru – About Mole Rats and Depression*, which was awarded as Best Game Beyond Entertainment in 2023 (Twisted Ramble Games, 2023). This digital game aims to inform about depression, to clarify common misunderstandings, and to convey how one can approach the topic depression or people suffering from depression. The game contains various sound elements, while communication between characters takes place via illustrations and symbols in speech bubbles. Details on the creation of the gaming videos including the presented content and story can be found in the Supplementary material.

### 2.4 Measures

To support reflection on and consolidation of the digital game’s content, we asked the participants after each gaming video “What aspects have left a lasting impression on you? Please name up to three key aspects that you remember.”

#### 2.4.1 Learning outcomes (RQ1)

After watching all three videos, participants indicated what they have learned from the digital game (RQ1a) based on the item “What did you learn from the digital game shown in the videos? Please name up to three points.” To measure what effects the digital game has had on the participants (RQ1b) and what effects participants think the digital game could have on others (RQ1c), they were asked “What effect does the game shown in the videos have on you? Please elaborate your thoughts in 2–3 bullet points” and “What effect could the game shown in the videos have on other people? Please elaborate your thoughts in 2–3 bullet points,” respectively.

#### 2.4.2 Learning motivation (RQ2)

We assessed learning motivation by adapting 15 items from Rüth and Kaspar (2021) that cover all components of the ARCS-V model. All items were adapted to the topic depression (e.g., original item: “How important to you is learning about evolution?”; adapted item: “It is important for me to learn something about the topic depression.”). Answers were given on a seven-point scale from 1 (*completely disagree*) to 7 (*completely agree*). The items were grouped into three thematic dimensions, and principal component analysis supported one-factorial structure of each dimension with eigenvalues ≥ 1, Kaiser-Meyer-Olkin measures of sampling adequacy > 0.74, and significant Bartlett’s tests of sphericity (all *p*s < 0.001) (see Supplementary Table S2), so that mean values were calculated: The first dimension (Cronbach’s *α* = 0.84) measures participants’ conviction that such games can be an interesting and relevant medium to learn about depression (RQ2a), covering one item on topic interest, two items on topic relevance (for oneself and others), and one item on confidence. The second dimension (*α* = 0.92) measures participants’ general learning motivation regarding the topic depression via three items focusing on volition (RQ2b). The third dimension (*α* = 0.93) measures participants’ satisfaction with the game at hand as a learning medium (RQ2c) with one item each for game recommendation, game continuance, game acquisition, game interest, game graphics, game sounds, an overall game rating, and the game’s appropriateness for learning about depression.

#### 2.4.3 Narrative engagement (RQ3)

Narrative engagement (RQ3) with the digital game was examined via the validated narrative engagement scale (Busselle and Bilandzic, 2009). We translated the 12 original items into German using translation-back translation (Brislin, 1970) and used the original seven-point scale that ranges from 1 (*completely disagree*) to 7 (*completely agree*).

#### 2.4.4 Depression stigma and depression literacy (RQ4)

Participants’ personal and perceived depression stigma was measured via nine items each from the German version of the Depression Stigma Scale (DSS) (Dietrich et al., 2014), using a five-point scale ranging from 1 (*completely disagree*) to 5 (*completely agree*). The DSS is a validated instrument with good construct validity as indicated by factor analysis (Griffiths et al., 2008).

Depression literacy was measured using the German version of the Depression Literacy Scale (Freitag et al., 2017), which is based on the original scale developed by Griffiths et al. (2004). The instrument contains 22 statements about Depression and allows to respond with “*correct*,” “*incorrect*” or “*do not know*.” Eight statements are correct in terms of describing depressive symptoms, and 14 statements are incorrect in terms of describing other symptoms. A higher number of correctly assigned statements indicates higher depression literacy.

### 2.5 Data analysis

To examine participants’ learning outcomes of the game (RQ1a-c), we created category systems using inductive content analysis (Mayring, 2022). For each learning outcome, about half of the data were used to create a first category system that was revised based on the full data set. A statement of a certain type had to be mentioned at least five times to form its own category. Two raters used the final category system to independently assign each statement to one of the categories. The inter-coder agreement was very good across all category systems with Cohen’s *κ* ≥ 0.74, indicating the appropriateness of the category systems (cf. Cohen, 1960). In the few cases in which there were disagreements between the coders, these were subsequently resolved through discussion, leading to a final and consensual assignment of the statements to the categories, which eventually enables frequency analyses (cf. Kaspar et al., 2014; Rüth et al., 2024).

Regarding learning motivation (RQ2a-c) and narrative engagement (RQ3), we calculated one-sample *t*-tests in addition to descriptive statistics to quantify the responses in terms of effect sizes.

To explore the role of the digital game in the context of destigmatization (RQ4), we calculated two linear regression models with personal and perceived depression stigma as dependent variables and seven independent variables: age, gender, depression literacy, learning motivation with its three dimensions (the conviction that such games can be an interesting and relevant medium to learn about depression, the general learning motivation regarding the topic depression, and the satisfaction with the game at hand as a learning medium), and narrative engagement. All quantitative analyses were performed using SPSS 30 (IBM, 2024).

## 3 Results

In response to the question “What aspects have left a lasting impression on you?,” which we asked after each gaming video, we found a total of *n* = 1,016 statements. In a nutshell, the participants named aspects related to the game’s goal, narrative, effects, characters, and design. They also reflected on social interactions displayed in the game and how they interpreted the game.

### 3.1 Learning outcomes (RQ1)

Regarding what participants have learned from watching the gaming videos (RQ1a), most statements were related to cognitive aspects (75.17%), followed by behavioral aspects (12.08%), emotional aspects (5.37%), aesthetic aspects (2.35%), and miscellaneous statements (1.34%). Some participants also stated to have learned nothing at all (3.69%). Concerning cognitive aspects, we found that watching the gaming videos provided participants information about the nature of depression (27.18%), that the individual environment is perceived as a relevant factor in the context of depression (18.46%), that communication with others appears to be important (11.07%), that participants gained specific information regarding the game’s narrative (10.07%), and that the game’s content elicited personal reflection processes (8.39%). All categories and example statements can be found in Table 1.

**Table 1:** Statements of participants on what they have learned from the digital game.

Categories and subcategories	Number of statements (*n* = 298)	Proportion (%)	Example statements
**Cognitive aspects**	224	75.17	
Information regarding the nature of depression	81	27.18	“Depression is something that stays with you for a long time, where there are good and bad phases,” “Terrible events affect everyone differently”
Relevance of the environment	55	18.46	“It’s easier with a friend,” “Doing what others are expecting from us”
Relevance of interpersonal communication	33	11.07	“Communication is important,” “Others are actually happy to help if you ask”
Information regarding the narrative	30	10.07	“community who tried to bring in yields but had to increase their work because of the caterpillars,” “The dog had a negative influence on the rodent’s thoughts and became stronger and bigger, when the rodent got worse.”
Personal reflection	25	8.39	“A reflection of one’s own unhealthy behavior patterns” “Although you can sometimes recognize from the outside that someone is not well at all or someone may even have depression, it is very difficult to really help from the outside, while you can do a lot of things wrong”
**Behavioral aspects**	36	12.08	“Do not always compare yourself with others,” “Try different things to come to a solution”
**Emotional aspects**	16	5.37	“It was stressful to spend time with,” “Develop sympathy or dislike for the characters”
**Aesthetic aspects**	7	2.35	“The game has very dark components at times,” “I learned that I like quiet tones and muted colors”
**No learning effects**	11	3.69	“Learned nothing,” “It really does not teach me anything”
**Miscellaneous**	4	1.34	“not that much, sometimes very incomprehensible,” “skills of the characters”

Regarding the specific effects of the game on participants (RQ1b), as shown by Table 2, a similar number of statements were related to cognitive effects (39.08%) and emotional effects (43.33%), while statements not describing clear effects were assigned to the miscellaneous category (17.59%). Cognitive effects of watching the game included insights (17.59%), incomprehension (8.14%), self-reflection (4.23%), thoughtfulness (4.23%), curiosity (2.61%), and specific thoughts about psychoeducation/depression (2.28%). Emotional effects included some rarely mentioned positive (5.86%) and negative emotions (5.86%) of different types, and the more frequently mentioned emotions sadness (7.82%), stress/strain (6.19%), anxiety (5.21%), boredom (3.26%), relaxation (3.26%), empathy (2.61%), anger (1.63%), and compassion (1.63%).

**Table 2:** Statements of participants on what effects the digital game has had on them.

Categories and subcategories	Number of statements (*n* = 307)	Proportion (%)	Example statements
**Cognitive effects**	120	39.08	
Insights	54	17.59	“Problems are often in your own head,” “It was about interpersonal relationships and the potential difficulties”
Incomprehension	25	8.14	“Not making sense,” “The game seems very complex and often unclear”
Self-reflection	13	4.23	“It could bring their own dissatisfaction with social aspects to the fore and lead to social comparison processes being reinforced,” “That I will pay more attention to my fellow human beings”
Thoughtfulness	13	4.23	“the video makes you think about whether people around you only say they are doing well when they are not.,” “thought-provoking: you think about whether there have similar situations in your own life”
Curiosity	8	2.61	“Interest in how the story ends and what it is actually about. Gave a lot of room for interpretation,” “but generates interest”
Specific thoughts about psychoeducation/depression	7	2.28	“I was depressed to see what a serious illness depression is,” “Awareness and motivation that you can do something about depressive moods”
**Emotional effects**	133	43.33	
Positive emotions^a^	18	5.86	“Entertaining” “Courage to approach others”
Negative emotions^a^	18	5.86	“Negative emotions,” “Worsening mood”
Sadness	24	7.82	“Sad, hopeless at the end,” “Sadness watching the suffering”
Stress/strain	19	6.19	“distressing: due to the main character’s worries and the persistent nightmares,” “hectic”
Anxiety	16	5.21	“It’s scary that the monster does not go away and follows you everywhere,” “it’s scary and frightening”
Boredom	10	3.26	“boring since the main character spent a lot of time in bed,” “but boring in parts”
Relaxation	10	3.26	“but also cute and cosy,” “Relaxed”
Empathy	8	2.61	“help people understand what is going on inside some people,” “Sympathy overall through the situations depicted”
Anger	5	1.63	“annoying (you want the main character to accept help),” “Annoying”
Compassion	5	1.63	“sad story, compassion,” “it made me feel compassion”
**Miscellaneous**	54	17.59	“very colorful presentation,” “it could show that music can evoke different moods in people”

^a^These are collective categories for rarely mentioned emotions without their own category (i.e., <5 mentions).

Regarding potential effects of the game on others according to the participants (RQ1c), as shown by Table 3, most statements were about emotional effects (52.81%), followed by cognitive effects (43.32%), and miscellaneous statements (13.03%). Again, insights (14.79%) was the most frequently named cognitive effect, followed by specific thoughts about psychoeducation/depression (8.10%), incomprehension (6.69%), self-reflection (4.93%), curiosity (4.23%), thoughtfulness (2.82%), and identification (1.76%). Emotional effects included some rarely mentioned positive (2.11%) and negative emotions (6.69%), and the more frequently mentioned emotions stress/strain (6.34%), anxiety (5.63%), entertainment (5.63%), sadness (5.63%), boredom (4.58%), empathy (3.52%), compassion (1.76%), and relaxation (1.76%).

**Table 3:** Statements of participants on what effects they think the digital game could have on others.

Categories and subcategories	Number of statements (*n* = 284)	Proportion (%)	Example statements
**Cognitive effects**	123	43.32	
Insights	42	14.79	“Misconception why people do not accept help.,” “Sometimes you have to actively address people’s concerns”
Specific thoughts about psychoeducation/depression	23	8.10	“sensitize more to depression,” “teaching, revealing to others how people with negative thoughts/emotions behave”
Incomprehension	19	6.69	“people might not understand the main character,” “lack of understanding”
Self-reflection	14	4.93	“It could bring one’s own dissatisfactions in relation to social aspects into the spotlight and increase social comparison processes,” “Mirror for one’s own personality/way of thinking”
Curiosity	12	4.23	“people with more perseverance to find out what is behind the symbolic language may become curious about what the game is about,” “possibly arouses interest”
Thoughtfulness	8	2.82	“brooding,” “makes you think”
Identification	5	1.76	“The game might bring back memories for some people, as everyone has probably been in a similar situation before,” “Complete identification”
**Emotional effects**	124	52.81	
Positive emotions^a^	6	2.11	“Hope for improvement,” “Behavioral patterns and actions are questioned and evaluated, positive feelings may arise”
Negative emotions^a^	19	6.69	“Distrust of some of the characters,” “It could definitely bring them down emotionally”
Stress/strain	18	6.34	“It could trigger negative thoughts,” “The game could still be stressful because you feel the pressure to perform”
Anxiety	16	5.63	“scary,” “it might be scary for younger people as the illustrations are drawn really spookily”
Entertainment	16	5.63	“People might find it funny because of the characters,” “captivating”
Sadness	16	5.63	“depressing because the main character is depressed,” “sad”
Boredom	13	4.58	“It might bore teenagers as nothing action-packed happens,” “it could bore them”
Empathy	10	3.52	“for people who do not see themselves in it: empathy,” “better understanding”
Compassion	5	1.76	“commiserating,” “compassion”
Relaxation	5	1.76	“The game could be calming because of the music and the world it depicts,” “relaxed”
**Miscellaneous**	37	13.03	“cute,” “colorful play of colors,” “versatile”

^a^These are collective categories for rarely mentioned emotions without their own category (i.e., <5 mentions).

### 3.2 Learning motivation (RQ2) and narrative engagement (RQ3)

In addition to descriptive statistics, we calculated one-sample *t*-tests (two-sided) comparing the observed mean ratings with the scale’s midpoint of 4. The midpoint of the scale therefore indicates moderate agreement with the motivation-related statements. Participants reported strong conviction that such games can be an interesting and relevant medium to learn about depression (RQ2a) (*M* = 4.64, *SD* = 1.40, *d* = 0.46), and their general learning motivation regarding the topic depression was even higher (RQ2b) (*M* = 5.73, *SD* = 1.22, *d* = 1.42). Satisfaction with the game at hand as a learning medium was moderate (*M* = 4.07, *SD* = 1.53, *d* = 0.04) (RQ2c). Detailed statistics for all items are shown in the Supplementary Table S3. Moreover, the participants reported a moderate narrative engagement in total (RQ3) (*M* = 3.97, *SD* = 1.21, *d* = 0.03), but with pronounced understanding of the narrative and emotional engagement. Details are shown in the Supplementary Table S4.

### 3.3 Depression stigma (RQ4)

As presented in Table 4, participants’ personal depression stigma (what participants think about depression) showed moderate-to-large significant bivariate correlations with gender (*r* = −0.35; 0 = male, 1 = female), depression literacy (*r* = −0.40), general learning motivation regarding the topic depression (*r* = −0.53), and narrative engagement (*r* = −0.19). In contrast, participants’ perceived depression stigma (what participants think most other people think about depression) did not show significant bivariate correlations with the independent variables of the regression model (all *p*s > 0.05).

**Table 4:** Results of the multiple regression analyses for personal and perceived depression stigma.

Independent variables	Personal depression stigma (R^2^ = 0.51, *F* = 15.18, *p* < 0.001)			Perceived depression stigma (R^2^ = 0.07, *F* = 1.14, *p =* 0.345)		
	*r*	*β*	*p*	*r*	β	*p*
Age	0.07	0.02	0.827	−0.12	−0.14	0.126
Gender (0 = male, 1 = female)	−0.35***	−0.28	< 0.001	−0.18	−0.17	0.065
Depression literacy	−0.40***	−0.29	< 0.001	−0.12	−0.12	0.327
Conviction that such games can be an interesting and relevant medium to learn about depression	−0.10	0.21	0.107	0.05	0.11	0.517
General learning motivation regarding the topic depression	−0.53***	−0.51	< 0.001	−0.04	−0.05	0.732
Satisfaction with the game as a learning medium	−0.15	0.03	0.859	0.00	−0.02	0.891
Narrative engagement	−0.19*	−0.24	0.056	−0.03	−0.05	0.696

r = bivariate correlation between independent and dependent variable; *β* = standardized regression coefficients of multiple regression analysis and associated *p-*value (bootstrapped via 10,000 iterations, two-tailed), *p < 0.05, **p < 0.01, ***p < 0.001.

The multiple regression models showed that the independent variables could explain 51% of the variance in personal depression stigma (*p* < 0.001) and 7% of the variance in perceived depression stigma (*p* = 0.345). First, gender, depression literacy, and general learning motivation regarding the topic depression showed a significant negative relation to personal depression stigma. Second, no independent variable was significantly related to perceived depression stigma in the regression model, reflecting the results at the bivariate correlation level. For details, see Table 4.

Finally, for exploratory reasons, we calculated *t*-tests for independent samples, showing that participants who did not study psychology (*n* = 43) had a lower depression literacy than psychology students (*n* = 74), *t*(115) = 3.70, *p* < 0.001, *d* = 0.71, and a higher personal depression stigma, *t*(115) = 2.70, *p* = 0.008, *d* = 0.52. Still, there were no significant differences between these subgroups regarding the other independent variables of the regression models (all *p*s ≥ 0.344) and perceived depression stigma (*p* = 0.975).

## 4 Discussion

We examined the potential of digital games for psychoeducation and destigmatization of depression by means of watching gaming videos. We selected the digital game *Duru* that aims to raise awareness and to inform particularly people without depression about depression (Twisted Ramble Games, 2023). Our results illustrate that watching the gaming videos conveyed information regarding depression and the game’s narrative, and it stimulated participants to reflect on the relevance of environmental factors, interpersonal communication, and personal matters. The game’s effects on participants ranged from cognitive effects such as insights, incomprehension, thoughtfulness, self-reflection, curiosity, and specific thoughts about psychoeducation and depression, to emotional effects, whereby many negative emotions (e.g., sadness, stress/strain, anxiety, and boredom) but also some positive emotions like relaxation and empathy were evoked. In contrast, when participants were asked about potential effects of the game on others, the proportion of assumed cognitive effects was higher. Particularly, participants mentioned specific thoughts on psychoeducation and depression relatively more often when being asked about effects of the digital game on others than on themselves. Further, proportionally, emotional effects on participants themselves were mentioned less frequently than assumed emotional effects on other people. This may be a special form of the third-person effect, claiming that people tend to expect larger (here: qualitatively different) effects on others than on themselves (Sun et al., 2008). Although we only found moderate satisfaction with the digital game *Duru* as a learning medium, participants reported a strong conviction that such digital games can be an interesting and relevant medium to learn about depression, and they also reported an even stronger motivation to learn about depression. Moreover, participants reported a moderate narrative engagement with a strong understanding of the narrative and emotional engagement. At the bottom line, these findings show that digital games can indeed be a suitable medium to convey information about depression and, as objects of reflection, encourage reflection on personal issues and people with depression (cf. Rüth et al., 2022). This makes digital games an interesting tool for psychoeducation.

Regarding depression stigma, we found two important results: on the one hand, the proposed regression model explained 51% of variance in participants’ personal thoughts about depression (personal depression stigma), which is remarkable. In this context, males had a higher personal depression stigma than females, corroborating previous results on gender differences in stigmatization (Al-Shannaq et al., 2023; Dietrich et al., 2014). Moreover, personal depression stigma was negatively related to participants’ depression literacy, which underscores the importance of factual knowledge. Personal depression stigma was also negatively related to participants’ general learning motivation regarding the topic depression, indicating the critical role of personal volition. On the other hand, the present model only explained 7% of variance in participants’ beliefs about what most other people think about depression (perceived depression stigma), suggesting that the proposed model is suitable for the assessment of personal thoughts but not suitable with regard to the perceived socially cultivated perspective on depression.

### 4.1 Limitations and outlook

Our findings are related to some limitations. First, our learning outcomes provide information about the role of digital games in psychoeducation based on qualitative findings, which could be complemented by experimental studies in the future. Such experimental studies, with appropriate comparison groups, could examine the size of the learning effects of gaming videos in comparison with other approaches of psychoeducation. Quantitative approaches also allow considering and controlling potentially confounding variables such as interest in mental health. The present results on the effects of watching gaming videos provide an ideal basis for the selection and operationalization of relevant constructs.

Second, we identified several variables of interest for depression stigma and could explain a substantial amount of variance in personal depression stigma, yet future research could investigate the role of other constructs. In addition, experimental and longitudinal studies are needed to investigate how the process of watching gaming videos causally influences stigmatization.

Third, it is still unclear whether the effects of watching gaming videos found here differ from the possible effects of actually playing digital games on learning outcomes and learning motivation. In principle, it should be noted that sensorimotor skills, personal effort and possibly also interaction with other players can represent initial hurdles for actual gaming. This is probably why watching games is such a popular activity that can also be engaging, whereas a lack of interactivity in terms of game controls could as well be considered as a freeing up of cognitive resources for learning from and reflecting on the game’s narrative. Regarding our study, the game’s narrative is deterministic so that interactivity in terms of making narrative choices might have played a relatively minor role. Notably, participants were instructed to take notes when watching the gaming videos and to reflect on aspects after each video that left a lasting impression on them, which could provide a starting point for future interventions.

Fourth, our findings are based on a convenience sample, with the majority of participants being psychology students who had a higher depression literacy and a lower personal depression stigma than the remainder of the sample. Still, the conviction that such digital games can be an interesting and relevant medium to learn about depression, the general learning motivation regarding the topic depression, and the satisfaction with the digital game as a learning medium did not differ between psychology students and other participants. Also, experienced narrative engagement in the group of psychology students was not different from the remainder of the sample. Accordingly, our approach of using gaming videos seems to work comparably well for the group of psychology students, despite different prerequisites. This is an important result, as several studies have emphasized that stigmatization is also a common problem among psychology students (e.g., Bannatyne and Stapleton, 2017; Pingani et al., 2021). As people in this group are often themselves later providers of psychoeducational methods and should show as little personal stigma as possible, the approach chosen here using digital games seems particularly promising for this group. However, only a few studies have yet investigated destigmatization in psychology students, for example, via clinical psychology lessons (Pingani et al., 2021) or game-based approaches (Mullor et al., 2019). Based on the qualitative data of the present study, we can only speculate whether watching digital games can have even stronger learning effects on groups with less prior psychological knowledge or related interests. This remains to be shown in future studies.

Finally, future research could consider several theories regarding psychoeducation, (de)stigmatization, and digital games (e.g., Krath et al., 2021; Thornicroft et al., 2022) to identify key constructs and to further unravel the role of digital games in psychoeducation and destigmatization of depression and other mental health problems in the future. This study has taken an important first step in this direction by broadening the perspective on the repertoire of intervention options in the contexts of psychoeducation and destigmatization.

## Data Availability

The original contributions presented in the study are included in the article/Supplementary material, further inquiries can be directed to the corresponding author.
