# Supplementary material for: Learning about depression by watching gaming videos: a case study on the potential of digital games for psychoeducation and destigmatization
Source: Front Psychol. 2025 May 26;16:1585571. doi: 10.3389/fpsyg.2025.1585571 (PMC12146388; doi:10.3389/fpsyg.2025.1585571)
Supplement: Supplementary file 1 [file Data_Sheet_1.pdf]

Supplementary Material

Learning about depression by watching gaming videos: A case study on the potential of digital games for psychoeducation and destigmatization

Marco Rüth, Raoul Bachmayer, Kai Kaspar

The gaming videos in our study were a compilation of screen recordings of the original digital game *Duru* (Twisted Ramble Games, 2023). We recorded mainly cutscenes to convey the narrative of the digital game. Some video sections also contain gameplay, so that participants could get an impression of the overall digital game. There was no further editing and no commentary, and it was not possible to influence the course of events in the digital game. For more information on the digital game, see https://www.twisted-ramble.com/press-kit-duru/

1 Overview of the content of the gaming videos

Content of gaming video 1 (7:28 min):

Tuli has a dream. Tuli explores their dream world. One can see large gray mole statues. The game dynamics (running, jumping) can be explored. Suddenly the sound changes. Tuli is being chased by a dark cloud with big and threatening teeth. Tuli wakes up.

Life in the gray mole community begins.

Tuli is invited for a meal by a friend. But first Tuli wants to see their best friend Lalele. Tuli tells Lalele about their nightmare. Lalele listens to Tuli and encourages Tuli. The two of them go out for a meal. Tuli sets off to work: collecting turnips.

Tuli takes the collected turnips to the warehouse. The supervisor is angry that Tuli has not collected more turnips.

Tuli exchanges ideas with their friends over a meal. The others express their negative feelings towards the supervisor.

At night, Tuli is woken up by a friend. The turnip store has been invaded by worms. Both go to the turnip warehouse.

The leading gray mole addresses the other gray moles. The warehouse is empty. Food is running out. Everyone has to stick together and everyone has to work harder.

While eating, Tuli talks to their friends again. Tuli decides to collect more turnips. Tuli then heads off to find turnips (2 minutes of gameplay follow).

<u>Content of gaming video 2 (5:59 min):</u>

Tuli is collecting turnips. While Tuli is looking for turnips, Bel, the metaphor for Tuli's depression, appears. Bel follows Tuli. Tuli cannot get away from Bel.

That night, Tuli takes their collected turnips to the supervisor. Tuli is worried that others might see Bel. Tuli feels drained and wants to work even harder. A friend brings Tuli food before Tuli sets off again to find turnips.

When Tuli arrives at the turnip warehouse, Tuli feels bad again because of having collected fewer turnips than the others. When they eat together, Tuli cannot eat anything because of their emotional state.

Tuli goes to sleep but is kept awake by Bel. The next day, Tuli is very exhausted. Tuli wants to be close to their friends.

After a meal, Tuli goes to the well with their friends. At the well, they meet other turnip collectors who notice that Tuli is not doing well. They give Tuli suggestions on what Tuli could do better: go out into nature, exercise, or eat well. Tuli feels bad and goes home.

<u>Content of gaming video 3 (9:06 min):</u>

Tuli has another dream. It reflects Tuli's restlessness and inner turmoil. In the end, Tuli is again haunted by the dark cloud. Tuli wonders whether it might have something to do with Bel. Tuli goes to Lalele to seek advice. Lalele is interested in how Tuli is feeling. However, Tuli is unable to open up to Lalele because Tuli is afraid Lalele will think Tuli is crazy. Lalele suggests Tuli to talk to the eldest gray mull. Tuli refuses and says goodbye. Tuli feels bad and thinks that no one wants to see them anymore.

At home, Tuli is again haunted by the thought that they are not giving their best. Tuli thinks that they are a burden to the others. Tuli looks in the mirror and sees how tired they look. Lalele knocks on Tuli's door in the morning and wants to pick up Tuli for lunch. Lalele offers to talk to Tuli if they want to. The two hug and Bel shrinks so that it fits into Tuli's backpack. Tuli goes in search for turnips (gameplay).

When Tuli arrives at the camp, they meet a friend who invites them to the well. Tuli agrees and the friend is pleased. At the well, Tuli sits a little apart again. The others wonder if Tuli is ill. The friend who invited Tuli suggests Tuli to take a vacation. Tuli has a guilty conscience and Bel grows again. This time Bel is larger than before. Tuli leaves and the others wonder what happened to Tuli. Lalele defends Tuli. Back home, Tuli is again worried about what the others think of them. Bel intensifies this carousel of thoughts. Tuli wakes up at night and sees how tired they look. Tuli thinks that nobody wants to have anything to do with them anymore.

Tuli has another dream. In their dream, Tuli finds a smiling mask, which Tuli puts on. The black cloud follows Tuli again and Tuli wakes up with the mask in their hand. Then, Tuli puts on the mask so that the others cannot see how unwell Tuli is. The others do not seem to notice anything at the meal. Tuli is complimented on how good they look. But Tuli soon realizes that wearing the mask is just a pretense.

2 Supplementary data

Table S1. Overview of sample characteristics.

**Characteristic**	**Response**	**Frequency (n = 117)**	**Proportion**
Age	M = 26.49 (SD = 10.44), range: 18–64 years		
Gender	Female	81	69.23%
	Male	31	26.50%
	Diverse	5	4.27%
Course of studies	Psychology	74	63.25%
	Other (e.g., business administration, social sciences, medicine)	19	16.24%
	None	24	20.51%
Courses on clinical psychology	Yes	25<br>(25)	21.37%<br>(33.78%)
	No	92<br>(49)	78.63%<br>(66.22%)
Educational background	University entrance qualification	80	68.37%
	Bachelor's degree	16	13.68%
	Master's degree	9	7.69%
	Completed traineeship	6	5.13%
	Doctoral degree	1	0.85%
	Graduate engineer	2	1.71%
	State examination	1	0.85%
	General certificate of secondary education	1	0.85%
	Completion of compulsory basic secondary schooling	1	0.85%

Note: Regarding courses on clinical psychology, frequency and proportions in brackets provide information for the subsample of psychology students.

Table S2. Principal Component Analysis (PCA) including factor loadings for participants' learning motivation with a digital game about depression.

	**Factor Loadings**		
**Items**	**Conviction that such games can be an interesting and relevant medium to learn about depression**	**General learning motivation regarding the topic depression (volition)**	**Satisfaction with the game as a learning medium**
1. Such games about the topic depression are interesting.	0.90		
2. Such games about the topic depression are relevant to me.	0.75		
3. Such games about the topic depression are relevant to others.	0.81		
4. Such games allow me to learn about the topic depression.	0.85		
5. It is important for me to learn something about the topic depression.		0.91	
6. I would like to learn something about the topic depression.		0.95	
7. It am motivated to learn something about the topic depression.		0.93	
8. I would recommend the game to others.			0.79
9. I would like to play the game in my leisure time.			0.83
10. I would be interested in having my own copy of the game.			0.84
11. This game is interesting.			0.88
12. I like the graphics in the game.			0.75
13. I like the sounds in the game.			0.73
14. I like the game overall.			0.94
15. This game is appropriate for learning about depression.			0.77
KMO	0.785	0.746	0.866
Bartlett's test	$p < 0.001$	$p < 0.001$	$p < 0.001$
Cronbach's α	0.84	0.92	0.93

Table S3. Participants' learning motivation with a digital game about depression.

**Measure**<br>Items	*M* (*SD*)	*d*
**Conviction that such games can be an interesting and relevant medium to learn about depression** (α = 0.84)	4.64***<br>(1.40)	0.46
Interest<br>Such games about the topic depression are interesting.	4.94***<br>(1.70)	0.55
Relevance (for oneself)<br>Such games about the topic depression are relevant to me.	3.65*<br>(1.93)	0.23
Relevance (for others)<br>Such games about the topic depression are relevant to others.	5.17***<br>(1.33)	0.88
Confidence<br>Such games allow me to learn about the topic depression.	4.90***<br>(1.78)	0.50
**General learning motivation regarding the topic depression (volition)** (α = 0.92)	5.73***<br>(1.22)	1.42
It is important for me to learn something about the topic depression.	5.92***<br>(1.23)	1.57
I would like to learn something about the topic depression.	5.78***<br>(1.33)	1.33
I am motivated to learn something about the topic depression.	5.49***<br>(1.36)	1.10
**Satisfaction with the game as a learning medium** (α = 0.93)	4.07<br>(1.53)	0.04
Game recommendation<br>I would recommend the game to others.	4.14<br>(1.84)	0.07
Game continuance<br>I would like to play the game in my leisure time.	2.86***<br>(1.81)	0.62
Game acquisition<br>I would be interested in having my own copy of the game.	3.15***<br>(1.92)	0.45
Game interest<br>This game is interesting.	4.42*<br>(1.81)	0.23
Game graphics<br>I like the graphics in the game.	4.40*<br>(1.98)	0.20
Game sounds<br>I like the sounds in the game.	4.52**<br>(2.05)	0.25
Overall game rating<br>I like the game overall.	4.23<br>(1.77)	0.13
Game's appropriateness for learning about depression<br>This game is appropriate for learning about depression.	4.82***<br>(1.81)	0.45

Note: Values depict mean (standard deviation) and effect size (Cohen's *d*). Asterisks indicate the results of one sample *t*-tests (two-sided) comparing the mean with the scale's midpoint of 4.
*$p < 0.05$. **$p < 0.01$. ***$p < 0.001$.

Table S4. Participants' narrative engagement (full scale) and its four subscales.

Measure<br>Items	*M* (*SD*)	*d*
**Narrative engagement** (α = 0.91)<br>Full scale (12 items, see below)	3.97 (1.21)	0.03
**Narrative understanding** (α = 0.84)<br>At some points, I found it difficult to understand what was going on in the video.<br>The meaning of the characters is unclear to me.<br>I found it difficult to recognize the thread of the story.	4.47*** (1.50)	0.32
**Attentional focus** (α = 0.90)<br>My mind wandered during the video.<br>I was thinking about other things during the video.<br>I found it difficult to focus on the video.	3.63* (1.64)	0.22
**Narrative presence** (α = 0.76)<br>During the video, I was physically in the room, but mentally I was in the world created by the story.<br>The video created a new world, which then suddenly disappeared when the video ended.<br>At times, the world of the story in the video was closer to me than the real world.	3.37*** (1.47)	0.43
**Emotional engagement** (α = 0.85)<br>The story affected me emotionally.<br>When the main character succeeded, I was happy, and when he suffered in some way, I was sad.<br>I felt sorry for some of the characters in the video.	4.62*** (1.49)	0.42

Note: Values depict mean (standard deviation) and effect size (Cohen's *d*). Asterisks indicate the results of one sample *t*-tests (two-sided) comparing the mean with the scale's midpoint of 4.
*$p < 0.05$. **$p < 0.01$. ***$p < 0.001$.
